# Direct cooling of the catheter tip increases safety for CMR-guided electrophysiological procedures

**DOI:** 10.1186/1532-429X-14-12

**Published:** 2012-02-01

**Authors:** Theresa Reiter, Daniel Gensler, Oliver Ritter, Ingo Weiss, Wolfgang Geistert, Ralf Kaufmann, Sabine Hoffmeister, Michael T Friedrich, Stefan Wintzheimer, Markus Düring, Peter Nordbeck, Peter M Jakob, Mark E Ladd, Harald H Quick, Wolfgang R Bauer

**Affiliations:** 1 Department of Internal Medicine I, University Hospital Wuerzburg, Wuerzburg, Germany; 2 Department of Experimental Physics V, University of Wuerzburg, Wuerzburg, Germany; 3 Biotronik SE & Co. KG, Berlin, Germany; 4 Vascomed GmbH, Binzen, Germany; 5 Erwin L. Hahn Institute for Magnetic Resonance Imaging, University of Duisburg-Essen, Essen, Germany; 6 Department of Diagnostic and Interventional Radiology and Neuroradiology, University Hospital, Essen, Essen, Germany; 7 Institute of Medical Physics (IMP), University of Erlangen-Nuernberg, Erlangen, Germany

**Keywords:** MRI, ablation, safety, catheter tip, EP Procedures, MR guidance

## Abstract

**Background:**

One of the safety concerns when performing electrophysiological (EP) procedures under magnetic resonance (MR) guidance is the risk of passive tissue heating due to the EP catheter being exposed to the radiofrequency (RF) field of the RF transmitting body coil. Ablation procedures that use catheters with irrigated tips are well established therapeutic options for the treatment of cardiac arrhythmias and when used in a modified mode might offer an additional system for suppressing passive catheter heating.

**Methods:**

A two-step approach was chosen. Firstly, tests on passive catheter heating were performed in a 1.5 T Avanto system (Siemens Healthcare Sector, Erlangen, Germany) using a ASTM Phantom in order to determine a possible maximum temperature rise. Secondly, a phantom was designed for simulation of the interface between blood and the vascular wall. The MR-RF induced temperature rise was simulated by catheter tip heating via a standard ablation generator. Power levels from 1 to 6 W were selected. Ablation duration was 120 s with no tip irrigation during the first 60 s and irrigation at rates from 2 ml/min to 35 ml/min for the remaining 60 s (Biotronik Qiona Pump, Berlin, Germany). The temperature was measured with fluoroscopic sensors (Luxtron, Santa Barbara, CA, USA) at a distance of 0 mm, 2 mm, 4 mm, and 6 mm from the catheter tip.

**Results:**

A maximum temperature rise of 22.4°C at the catheter tip was documented in the MR scanner. This temperature rise is equivalent to the heating effect of an ablator's power output of 6 W at a contact force of the weight of 90 g (0.883 N). The catheter tip irrigation was able to limit the temperature rise to less than 2°C for the majority of examined power levels, and for all examined power levels the residual temperature rise was less than 8°C.

**Conclusion:**

Up to a maximum of 22.4°C, the temperature rise at the tissue surface can be entirely suppressed by using the catheter's own irrigation system. The irrigated tip system can be used to increase MR safety of EP catheters by suppressing the effects of unwanted passive catheter heating due to RF exposure from the MR scanner.

## Background

Electrophysiological (EP) procedures are a well established method both for diagnostic characterization of the electrical activation of the human heart and for curative treatment of cardiac arrhythmias. X-ray fluoroscopy is used for imaging guidance. The therapeutic benefits are well documented, and EP procedures very often offer a permanently successful treatment option associated with few or no side effects. However, fluoroscopy allows only a very limited insight into the exact heart anatomy. Soft body tissue such as heart muscle, valvular structures, and vessels are only faintly projected unless calcifications have developed. During the procedure, the patient and electrophysiologist are both exposed to ionizing radiation. Cardiovascular magnetic resonance (CMR) could be a potential imaging alternative for procedure guidance. CMR offers enormous insight into organ structure and function without exposing the patient or investigator to ionizing radiation. The images are acquired with the help of strong static and gradient magnetic fields as well as radiofrequency (RF) fields.

However, it is known that long electrically conducting wires such as guide wires or catheters interact with the RF transmit field and couple with the electric field, consequently producing localized heating especially at the catheter tip [1,2]. Various strategies have been discussed to limit RF-induced instrument heating, such as the introduction of coaxial chokes or the use of highly resistive cables or microtransformers [3-5]. However, up to date, the realization of coaxial chokes or other means for suppression of heating that is induced by the MR scanner's RF field is still incomplete, and a risk of potential RF heating remains. Accordingly, an additional safety mechanism would be desirable that does not interfere with the electrophysiology measurements or ablation fields and would be easy to realize without the need for significant catheter design changes. The solution might be found in the EP catheter's own irrigation system for cooled-tip ablation. In this paper we investigate whether tip irrigation can be used to increase catheter safety independent of the surroundings or CMR sequences under use by suppressing RF-induced instrument heating below a threshold of optimally 2°C.

## Methods

The concept of the test setup is based on the fact that the thermal effects of a temperature rise at the catheter tip are independent of the causative energy source. Energy is deposited into the tissue via the catheter tip, which leads to a temperature rise within the tissue. The catheter tip itself does not change its temperature. The catheter shaft is coated with an insulating material, thus restricting most of the energy transmission from the catheter to the tissue to the uninsulated catheter portions (mostly the tip). The uninsulated portions of the catheter allow galvanic heating at the tip, which easily exceeds any possible heating effects along the insulated shaft and in fact will be dominate for this device. Tests prior to this work failed to show any, with regard to safety, relevant heating effects along the insulated shaft.

There is no difference between the heating that is caused by the RF field of the MRI and the heating that is produced with the help of an ablation generator because the resulting temperature rise is a superposition of causative energy sources (see additional file 1). This allows the utilization of a two-step approach. Tests in the MR scanner are used to evaluate the highest expected temperature rise at the catheter tip due to the electric field of the RF transmit body coil. A second set of tests uses the ablation generator as energy source for tissue heating and focuses on the catheter irrigation capabilities.

### Interface phantom

A special tissue interface phantom was designed for the tests. It consists of a fluid phase that represents the blood and a solid phase simulating the myocardial tissue wall. It is important to focus on the interface because of two main reasons: firstly, it simulates a realistic situation in a patient. Tissue damage due to induced RF catheter heating occurs at the interface between blood and vessel wall. Secondly, it allows a realistic evaluation of the irrigation system and more exact temperature measurements in the tissue. Irrigation tests in a one-phase phantom resembling those used in other studies would not be reliable [6,7]. In water, heat convection will eliminate a major part of the generated heat even without irrigation, and thus the irrigation effect will most likely be overestimated. On the other hand, phantoms filled with gel (e.g. HEC) show good suppression of heat convection. However, gel does not allow drainage of the irrigation fluid. Consequently, after the start of irrigation, a fluid bubble forms around the catheter tip. The conditions then approach a fluid phantom.

An accurate temperature measurement is dependent on an exact placement of the temperature sensor. This exact placement can be achieved with direct visual control. The use of ground meat for the solid phase of the phantom allows such an exact positioning, because ground meat is not a solid piece of muscle but can be formed flexibly. In unprocessed meat, information on the location of the sensors within the muscle, especially the distance to the surface and the alignment, would not be obtainable. The interface phantom's fluid phase is physiological saline solution. No simulation of blood flow was performed in order to examine the catheter's irrigation system independent of the body's own irrigation by blood flow. The test setup thus aims for a worst-case situation where no or minimal blood flow is present, for example in the atria during atrial fibrillation.

**Figure 1:**
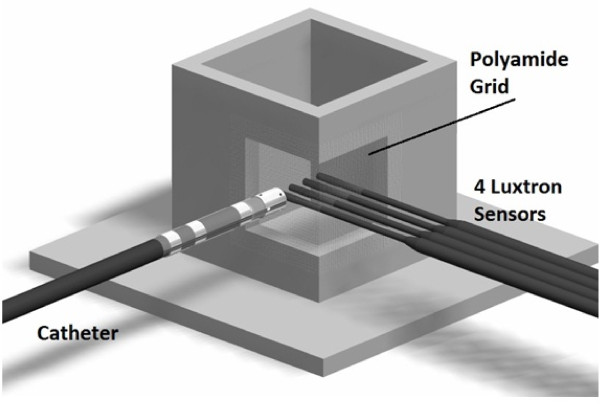
**Schematic view of the test cube**. The top face is left open to allow for filling with ground beef. The polyamide grid is placed firmly over the open side parts. The catheter is placed perpendicular to the polyamide grid. The polyamide grid on the neighboring side has a small slit allowing the temperature sensors to be inserted into the beef core. The sensors are placed parallel to each other and perpendicular to the catheter tip. The distances of the sensors to the catheter tip/polyamide grid surface are 0, 2, 4, and 6 mm.

The core part of the interface phantom is a 20 × 20 × 20 mm^3 ^cube (Figure 1). The top face and square holes in two bordering sides are open. The open side parts are covered with a polyamide grid (PA-100/32, Franz Eckert GmbH, Waldkirch, Germany) that forms a firm and tight surface with good permeability for water and no influence on electrical characteristics. The grid ensures identical test setups for all measurements. During the tests, the cube is filled with ground beef that is pressed firmly into the test cube. The same amount of beef was used for all tests. The beef had been minced thoroughly in order to ensure a homogenous texture, and a low fat content (< 15%) was chosen. Concerning the temperature and heating characteristics, processed meat resembles unprocessed meat, and processed meat has been used in previous studies that described thermal qualities and heating effects [8,9].

The catheter tip's position is perpendicular to the polyamide grid, and the tip directly touches the polyamide grid surface. The polyamide grid that covers the bordering side has a small gap that allows temperature sensors to be inserted into the beef filling. Four fluoroscopic sensors (Luxtron, Santa Barbara, CA, USA) were used. The tip of the first sensor was placed perpendicular to and directly neighboring the catheter tip, thus measuring the temperature at the surface [10]. The four sensors were placed at distances of 0, 2, 4, and 6 mm from the catheter tip. Eick et al. (2003) showed that tissue temperatures up to 70°C would create lesions with a maximum depth of less than 7 mm [11]. A tissue temperature of 70°C equals a temperature rise of 33°C above body temperature and is the target temperature during a therapeutic ablation. However, pretests in our working group showed that during catheter tip heating due to the MR scanner's RF energy, a temperature rise of less than 33°C is to be expected. Consequently, a temperature measurement at a maximum tissue depth of 6 mm was considered sufficient.

The interface phantom was used for both the evaluation of RF-induced catheter heating and the evaluation of the catheter's irrigation system. A standard open irrigation ablation catheter (AlCath Flux eXtra. VascoMed GmbH, Binzen, Germany) was modified by replacing all ferromagnetic components with non-ferromagnetic metals and high performance polymers. The changes in the components did not affect the ablation function. The details of the tip design remained unchanged.

### Tests in the MR scanner

The evaluation of the maximum RF-induced catheter heating uses the MR scanner's own RF field that is produced by the RF transmit body coil. A 1.5 T whole-body scanner (Magnetom Avanto, Siemens Healthcare, Erlangen, Germany) was used. No imaging sequences were applied. The applied sequence was a modified inversion recovery (IR) snapshot FLASH sequence that can be used to monitor temperature based on T1 changes (not used here) [12]. This sequence was chosen because it has long duration and an easily adjustable SAR. Off-resonant high-power heating pulses at a frequency of 128 kHz below the Larmor frequency (64 MHz) were applied both during each TR cycle and the intermediate delay time between the components of the sequence. The specific absorption rate (SAR) is dependent on the power of the off-resonance RF pulses. The sequence parameters were: TR 6.0 ms, TE 3.4 ms, FOV 240 × 120 mm, slice thickness 5 mm, flip angle 8° for imaging, 142° for the off-resonant heating pulses, resolution 192 × 96, duration 7:14 min. The whole-body SAR as reported by the scanner software was 3.7 W/kg [13]. The sequence had a high SAR and a sufficiently long duration to allow the tissue temperature to reach an equilibrium level. The sequence as described would not be used in patients, but serves only for the evaluation of the tissue heating.

**Figure 2:**
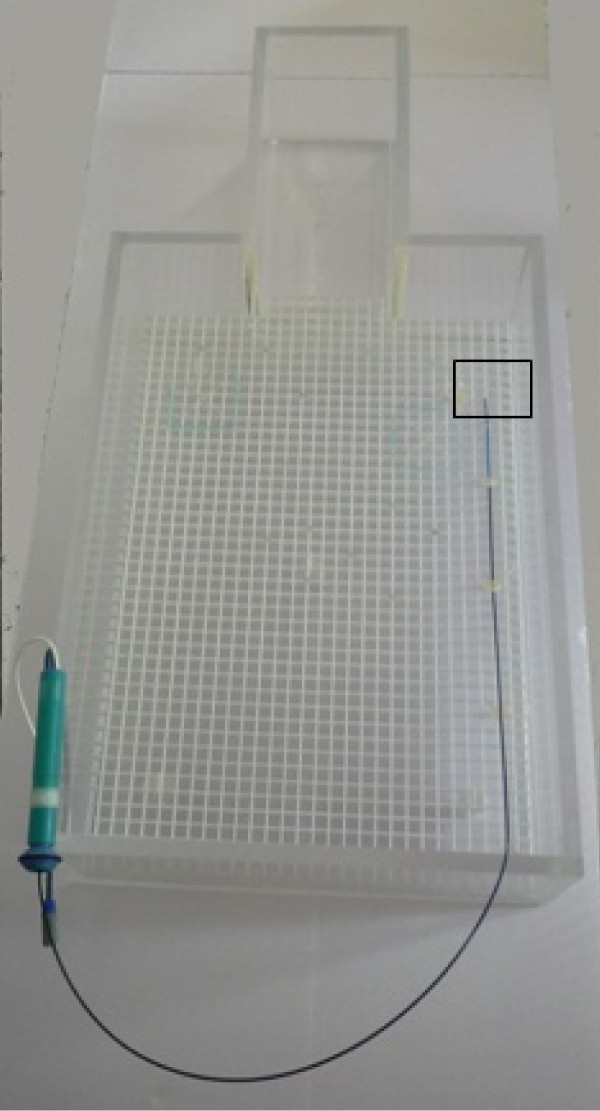
**Catheter pathway in the MR scanner**. The catheter is placed at the lateral left side of the phantom. The square indicates the location of the beef cube.

The tests in the MRI scanner were performed on a body phantom representing a body torso adapted from the ASTM (F2182_02b). The phantom consists of a rectangular body and a rectangular head section, simulating a simplified human body (body section 60.9 cm × 43.2 cm, head section 29.2 cm × 16.5 cm, height of both section 15.2 cm). The phantom was filled with 40 liters of isotonic saline solution which was kept at room temperature (19 - 23°C). The interface phantom was placed inside the body phantom. The relevant electric fields that can cause catheter tip heating are not distributed evenly in the phantom. The highest electric fields are to be expected at the lateral walls of the phantom. Accordingly, the highest temperature rise due to RF energy is to be expected when the catheter is placed within these areas. The catheter was placed in a straight line along the left lateral wall of the body section of the phantom, at a distance of 2 cm from the wall and 7 cm above the phantom bottom (Figure 2) [3,14,15]. Other more medial positions of the catheter in the phantom as well as other placements of the catheter handle were tested but showed less heating. In an attempt to determine the worst possible heating effect, this non-anatomical position was chosen.

### Tests with the ablation generator

A standard ablator (Osypka HAT 300, Rheinfeld, Germany) was used for the evaluation of the irrigation capabilities. The ablator was run in the power-controlled mode. This mode defines a fixed power level that is maintained throughout the entire ablation duration and is independent of the temperature that is measured with the catheter's thermocouple system. A safety cutoff temperature of 96°C is part of the standard programming of the ablator. The rationale behind choosing such a set up is that in the temperature-controlled mode, the effects of the irrigation system may remain hidden. In a certain range of target temperatures the ablator will adjust the power level to higher values to maintain the target temperature, thus suppressing the irrigation effect. The chosen power output of the ablator was 1-6 Watts and well below the typical therapeutic settings (up to 75 W). Preliminary tests showed that the temperature obtained with these power levels corresponds very well to the temperature range that was obtained by using MR RF energy in the test setup. A unipolar ablation mode was chosen. The required indifferent electrode was placed at the lateral wall of the water basin and was kept in contact with the fluid phase.

The duration of the ablation was set at 2 min, with no irrigation present during the first minute and an immediate stop of irrigation at ablation cessation. A Qiona pump (Biotronik SE & Co. KG, Berlin, Germany) was used. The lowest irrigation rate used was 2 ml/min, the highest evaluated irrigation rate was 35 ml/min. Taking the considerable volume of 2100 ml/h for an irrigation rate of 35 ml/min into account, irrigation rates above this threshold were considered unacceptable for patient procedures. The suppression of the temperature rise during ablation was considered successful when a temperature rise of not more than 2°C was achieved, and as far as possible, flow rates were adjusted accordingly. However, considering studies from other groups that have shown noticeable and reversible changes in the cellular excitability and automaticity of paced muscles at temperatures above 45°C at a median ablation time of 60 sec, a maximum temperature rise of 8°C was still considered acceptable [16,17].

The tests with the ablator were performed in a rectangular water basin filled with 9 L saline solution at body temperature.

### Contact force

The contact between the catheter tip and the tissue is an important factor for the created lesion size and can be characterized by the contact force between the catheter tip and the tissue. A few previous studies have focused on the effects of the contact force on the lesion size and showed that the lesion size increases linearly with the contact force. The contact force also has an effect on the occurrence of adverse events such as steam pops and charring. When focusing on the therapeutic relevance of the contact force, prior works have used contact forces equivalent to the weight of masses between 2 and 40 g [18-20]. Mean contact forces for atrial flutter ablation procedures are around the weight of a mass of 7 - 37 g [21]. However, these contact forces are used for therapeutic ablation. In our setup, we do not focus on lesion creation for a therapeutic ablation, but on unwanted and unpredicted lesion creation due to RF-induced catheter heating. The chosen weight range is wide in order to simulate two extreme situations - a simple resting of the catheter against the vessel wall resulting in a very low contact pressure (10 g) and a possible trapping of the catheter against the vessel wall (90 g). In our test setup, the contact force was measured with a contact force gauge (PCD-FM50, PCE GmbH, Arnsberg, Germany).

## Results

### RF-induced catheter heating in the MR scanner

**Figure 3:**
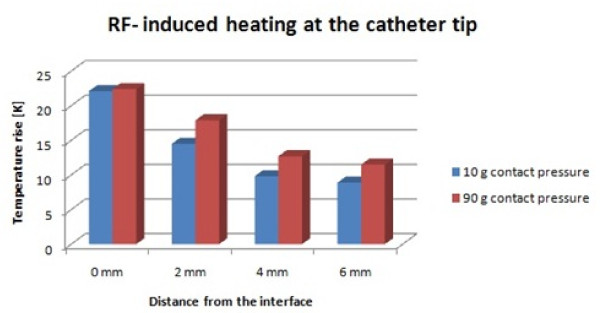
**MR-RF induced heating at the catheter tip**. The difference in the temperature rise at different contact pressures increases with tissue depth.

The temperature rise was highest directly at the contact area between catheter tip and the tissue surface. The temperature rise decreased with increasing tissue depth, as expected. The two examined contact pressures, equaling the weight of the masses 10 g and 90 g, did not show an influence on the temperature rise at the surface. The temperature rises that were measured were 22.1°C at the weight of the mass 1 g and 22.4°C at the weight of the mass 90 g. The temperature rise at a distance of 2 mm from the surface differed by 3°C depending on the contact pressure (14.5°C at 10 g and 17.9°C at 90 g) (Figure 3).

### Ablation-generator-induced catheter heating outside the MR scanner - evaluation of irrigation abilities

**Figure 4:**
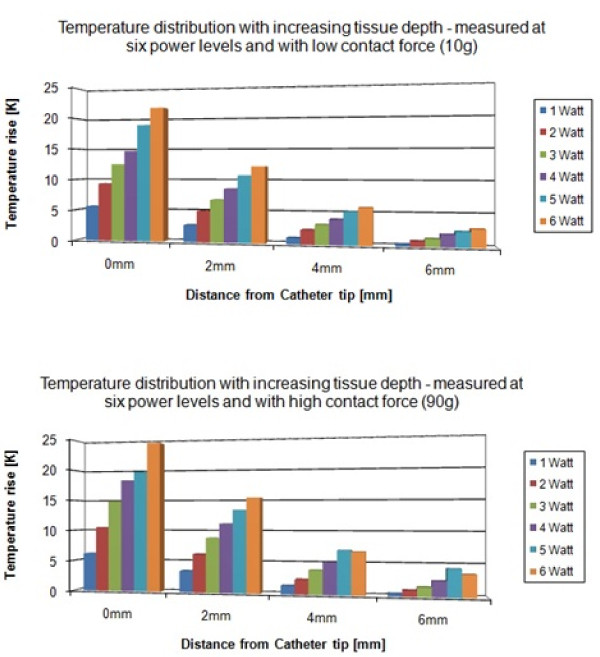
**Ablation generator induced temperature rises**. Temperature distribution measured at increasing tissue depth from the catheter tip. Different power levels were examined. The upper figure shows the temperature development at a low contact force. The lower figure shows a similar development of temperature that was measured at a high contact force. The distribution of heat is not significantly influenced by the contact pressure. Additionally, the temperature decline due to increasing distance from the catheter tip remains unchanged.

**Figure 5:**
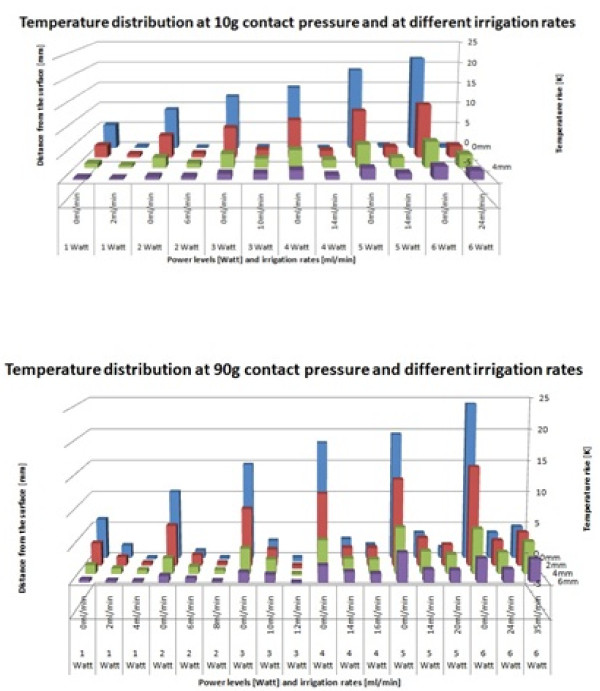
**Temperature distribution at different distances from the catheter tip: 0 mm, 2 mm, 4 mm, and 6 mm from the catheter tip and the interface surface**. Higher contact pressures require higher irrigation rates to achieve similar cooling effects. Each test started at the baseline temperature, e.g. the prior tests did not influence the following test results.

The electromagnetic power that causes the heating at the catheter tip is produced by the ablation generator and conducted into the tissue. Higher power levels created higher temperature rises at the interface (Figure 4). The MR-induced maximum heating correlated to the heating generated with 6 W of ablation power (21.6°C at 10 g contact force, 24.3°C at 90 g contact force). The applied contact pressure influenced the temperature rise at the interface only moderately (Figure 5). At increasing distance from the catheter tip, the temperature diminished regardless of contact pressure. Figure 5 also shows that the temperature rise was proportional to the ablation power level used. No charring or steam pops occurred.

The irrigation abilities of the catheter allowed - for the power levels from 1 to 6 W - nearly complete suppression of the generated temperature rise. The irrigation rate required to achieve a remaining temperature rise of no more than 2°C depends on the power level and consequently on the initial temperature rise. Higher power levels demand higher irrigation rates for successful cooling. The contact pressure also influenced the required irrigation. A contact pressure equaling the weight of a mass of 90 g required higher irrigation rates than a contact pressure equaling the weight of a mass of 10 g. For the power output of 1 W and 2 W, the irrigation rate had to be increased slightly (at 1 W from 2 ml/min to 4 ml/min, at 2 W from 6 ml/min to 8 ml/min) at higher contact pressure in order to suppress the temperature rise below the threshold of 2°C. At the power outputs of 5 W and 6 W, the required irrigation rate increased rapidly from 14 ml/min to 20 ml/min and from 24 ml/min to 35 ml/min for the two contact pressures. Suppression of the temperature rise to 2°C could not be achieved at 6 W and high contact pressure even for the maximum irrigation rate of 35 ml/min (Figure 5). Temperature rises created with power levels above 6 W showed increasing resistance toward cooling effects of the catheter's irrigation.

## Discussion

The tests in the MR scanner showed the MR-RF-induced heating at the catheter tip in a possible worst case situation where the catheter is brought into areas of high electric fields and no irrigation is present. Based on the distribution of the electric fields, changes in the catheter position would lead to a decrease in the temperature rise [14]. It has been shown in animal tests that while maneuvering the catheter a maximum temperature rise of 7.5°C can be reached [22]. In vivo the expected temperature rises seem to be much less than the maximum value in our MR tests. The maximum temperature rise of approximately 22.4°C shows that the MR RF field that is emitted by the MR scanner's body coil can cause tissue heating which easily exceeds the in vivo measurements. 22.4°C is well above a temperature rise of 14°C that will cause irreversible tissue damage [16,17]. However, the heating effect depends on various factors, e.g. the position within the electric field, and can quickly change. It is also very difficult to examine all possible positions of the catheter within its anatomical track. Thus, it is important to determine the maximum heating effect, regardless of the typical expected heating in vivo. The ASTM phantom was originally designed for the testing of implanted devices. It is a very simplistic version of a human torso. It is standardized, thus allowing defined test setups. By no means are all aspects of a human body represented, e.g. the vessel systems are lacking. Data from such phantom studies should not be transferred to use in patients without critical interpretation. However, the phantom features important aspects of the human body which are relevant for the RF-induced heating of both devices and catheters, such as physiologic dielectric properties, appropriate body volumes, and known electric field distributions. The lack of a vessel system and blood flow simulation cause a possible worst case situation ignoring perfusion.

The tests outside the MR scanner clearly showed that within the temperature range of 0 - 22°C temperature rise, the catheter's irrigation system sufficiently suppresses this heating effect. However, using a cooled-tip EP catheter to prevent lesion creation seemingly contradicts the known benefit of creating larger and deeper lesions. The irrigation decreases the likelihood of scarring and steam pops at the surface, thus allowing the use of higher power levels. When focusing on one fixed power output level, the irrigation reduces the lesion's diameter at the surface but not necessarily the volume and depth of the lesion [23]. This is especially true when therapeutic power levels are applied. However, low power levels create lesions that have a smaller maximum depth and so are more susceptible to cooling effects. We have also shown that the surface temperature can be reduced drastically, whereas the temperature increases within the tissue do not reach the same levels as the surface temperature, even without irrigation. The electrical conductivity of the irrigation solution can differ from the surrounding tissue; however, the tissue conductivity is not influenced by the irrigation [24].

The temperature threshold during the irrigation is defined both by safety standards and by works on the cellular effects of cardiac ablations. The safety standards define an optimal threshold of not more than 2°C temperature rise [25]. However, works on cardiac ablation clearly point out that irreversible and thus permanent damage to the cardiac muscle does not occur until a temperature rise around 14°C and a mostly reversible loss of excitability at a median temperature of 8°C [16,17]. The optimal residual temperature rise during irrigation in our tests was considered to be 2°C; however, temperature rises which remained below 8°C were still considered to be acceptable.

The tests in our MR scanner showed that the relevant power levels for the irrigation tests are well below the power levels that are usually used for generating therapeutic lesions. Consequently, the tissue depth of the created lesion is limited even when using no irrigation for cooling. Depending on the power level and the irrigation rate, irrigation leads to an almost complete suppression of tissue temperature rise both at and beyond the surface. When no irrigation is applied, the temperature rise is nearly uninfluenced by the contact pressure. However, the irrigation effect is modulated by the contact pressure, requiring higher irrigation rates for higher contact pressure at the same power output. The reason for this might lie with the decreased heat dissipation in the tissue area that is directly in contact with the catheter tip.

Animal studies showed a maximum heating of 7.5°C [22]. Further tests in animals showed no scarring or burns after an MR-guided procedure [26]. The heating in patients is most likely limited to similar temperature rises. Low irrigation rates of 6 ml/min or less might already be adequate to ensure the patient's safety. However, further tests and studies in animals are required before fixing a defined rate.

### Limitations

This work is a phantom study; consequently, there are limitations to the study based on the test setup and the chosen materials. Though important features of the anatomy (interface) were considered, not all anatomic aspects could be simulated adequately, e.g. blood flow and catheter pathway in a patient, thus provoking a limited worst-case situation. Despite these limitations, the setup allows a first valuable step toward evaluation of this concept, and further tests, e.g. in animals, are to be planned.

## Conclusion

Optimal visual overview and lack of ionizing radiation are two major advantages of CMR that would be desirable in EP procedures. However, mainly due to the requisite imaging RF field, the use of conventional catheters and instruments in CMR is limited, as RF catheter tip heating might pose a serious risk for the patient. Even for catheters which have been modified to make them more MR compatible, an additional safety system would be desirable to control the remaining risk of catheter tip RF heating. Here we focused on a new approach toward increased safety in CMR-guided EP procedures: use of the catheter's own irrigation system. We showed that the irrigation system removes the heat from the surface and consequently allows the suppression of the RF-induced tissue heating at the catheter tip. No additional means such as sheaths or CMR sequence modifications are needed, as this system is based solely on the catheter's own irrigation system. This technique is limited to suppressing a maximum temperature rise of roughly 22°C. Temperature rises above this value require irrigation rates exceeding 35 ml/min, which could lead to an unacceptable volume overload for the patient. Ultimately, the deposited energy exceeds the irrigation capability even at higher irrigation rates. Nevertheless, we have demonstrated that the standard irrigation abilities of an EP catheter can be considered sufficient to increase the safety for CMR-guided EP procedures.

## Competing interests

TR, DG, SW, MD, PN: none; PJ: consultant of Siemens; OR, WRB, MEL, HHQ: consultant of Biotronik; IW, SH, MTF: employees of Biotronik; WG, RK: employees of Vascomed

## Authors' contributions

TR developed the design of the experiments and used material, carried out all experiments, wrote the manuscript and merged all feedback from the co-authors into the final manuscript. DG developed the used MRI sequence, contributed to the test design and the experiments. OR, PN, PMJ contributed to the experiment design and revised the manuscript critically for intellectual content. IW, MEL, HHQ, WRB contributed to the test design, contributed to important intellectual content of the manuscript and revised it critically for intellectual content. WG, RK, SH, MTF contributed to the test and material design and revised the manuscript critically for intellectual content. SW, MD contributed to the design of test material and the study design. All authors read and approved the final manuscript.

## Supplementary Material

Additional file 1 **Impact of frequency on the power deposition at the catheter tip**. The power deposition at the catheter tip into the tissue is dependent on the wave propagation phenomena, distal reflexion and losses in the surrounding medium, and changes with the wave length. This impact of frequency on the power deposition at the catheter tip can be simulated numerically, explaining the rationale behind using two different energy sources (MRI and standard ablation generator) in our test setup. Click here for file

## References

[B1] Bassen H Kainz W Mendoza G Kellom T MRI-induced heating of selected thin wire metallic implants-- laboratory and computational studies-- findings and new questions raised Minim Invasive Ther Allied Technol 2006 15 2 76 84 10.1080/13645700600640931 16754190

[B2] Konings MK Bartels LW Smits HF Bakker CJ Heating around intravascular guidewires by resonating RF waves J Magn Reson Imaging 2000 12 1 79 85 10.1002/1522-2586(200007)12:1<79::AID-JMRI9>3.0.CO;2-T 10931567

[B3] Ladd ME Quick HH Reduction of resonant RF heating in intravascular catheters using coaxial chokes Magn Reson Med 2000 43 4 615 619 10.1002/(SICI)1522-2594(200004)43:4<615::AID-MRM19>3.0.CO;2-B 10748440

[B4] Settecase F Hetts SW Martin AJ Roberts TP Bernhardt AF Evans L Malba V Saeed M Arenson RL Kucharzyk W RF Heating of MRI-Assisted Catheter Steering Coils for Interventional MRI Acad Radiol 2011 18 3 277 285 10.1016/j.acra.2010.09.012 21075019 PMC3034801

[B5] Weiss S Wirtz D David B Krueger S Lips O Caulfield D Pedersen SF Bostock J Razavi R Schaeffter T In vivo evaluation and proof of radiofrequency safety of a novel diagnostic MR-electrophysiology catheter Magn Reson Med 2011 65 3 770 777 10.1002/mrm.22669 21337409 PMC3715087

[B6] Detti V Grenier D Perrin E Beuf O Assessment of radiofrequency self-heating around a metallic wire with MR T1-based thermometry Magn Reson Med 2011 10.1002/mrm.22834 21360744

[B7] Yeung CJ Karmarkar P McVeigh ER Minimizing RF heating of conducting wires in MRI Magn Reson Med 2007 58 5 1028 1034 10.1002/mrm.21410 17969097

[B8] Banerjee A Ogale AA Das D Mitra K Subramanian C Temperature Distribution in Different Materials Due to Short Pulse Laser Irradiation Heat Transfer Engineering 2005 26 8 41 49 10.1080/01457630591003754

[B9] Kangarlu A Ibrahim TS Shellock FG Effects of coil dimensions and field polarization on RF heating inside a head phantom Magn Reson Imaging 2005 23 1 53 60 10.1016/j.mri.2004.11.007 15733788

[B10] Mattei E Triventi M Calcagnini G Censi F Kainz W Bassen HI Bartolini P Temperature and SAR measurement errors in the evaluation of metallic linear structures heating during MRI using fluoroptic probes Phys Med Biol 2007 52 6 1633 1646 10.1088/0031-9155/52/6/006 17327653

[B11] Eick OJ Bierbaum D Tissue temperature-controlled radiofrequency ablation Pacing Clin Electrophysiol 2003 26 3 725 730 10.1046/j.1460-9592.2003.00123.x 12698673

[B12] Ehses P Fidler F Nordbeck P Pracht ED Warmuth M Jakob PM Bauer WR MRI thermometry: Fast mapping of RF-induced heating along conductive wires Magn Reson Med 2008 60 2 457 461 10.1002/mrm.21417 18570323

[B13] Gensler D Fidler F Ehses P Warmuth M Reiter T Duering M Ritter O Ladd ME Quick HH Jakob PM MR Safety: Fast T1 Thermometry of the RF- Induced Heating of Medical Devices Magn Reson Med 2011 10.1002/mrm.24171 22287286

[B14] Nordbeck P Fidler F Weiss I Warmuth M Friedrich MT Ehses P Geistert W Ritter O Jakob PM Ladd ME Spatial distribution of RF-induced E-fields and implant heating in MRI Magn Reson Med 2008 60 2 312 319 10.1002/mrm.21475 18666101

[B15] Wildermuth S Dumoulin CL Pfammatter T Maier SE Hofmann E Debatin JF MR-guided percutaneous angioplasty: assessment of tracking safety, catheter handling and functionality Cardiovasc Intervent Radiol 1998 21 5 404 410 10.1007/s002709900288 9853147

[B16] Nath S Lynch C Whayne JG Haines DE Cellular electrophysiological effects of hyperthermia on isolated guinea pig papillary muscle. Implications for catheter ablation Circulation 1993 88 4 Pt 1 1826 1831 8403328 10.1161/01.cir.88.4.1826

[B17] Wood M Goldberg S Lau M Goel A Alexander D Han F Feinstein S Direct Measurement of the Lethal Isotherm for Radiofrequency Ablation of Myocardial Tissue Circ Arrhythm Electrophysiol 2011 4 3 373 8 10.1161/CIRCEP.110.961169 21406684

[B18] Shah DC Lambert H Nakagawa H Langenkamp A Aeby N Leo G Area under the real-time contact force curve (force-time integral) predicts radiofrequency lesion size in an in vitro contractile model J Cardiovasc Electrophysiol 2010 21 9 1038 1043 10.1111/j.1540-8167.2010.01750.x 20367658

[B19] Thiagalingam A D'Avila A Foley L Guerrero JL Lambert H Leo G Ruskin JN Reddy VY Importance of catheter contact force during irrigated radiofrequency ablation: evaluation in a porcine ex vivo model using a force-sensing catheter J Cardiovasc Electrophysiol 2010 21 7 806 811 20132400 10.1111/j.1540-8167.2009.01693.x

[B20] Yokoyama K Nakagawa H Shah DC Lambert H Leo G Aeby N Ikeda A Pitha JV Sharma T Lazzara R Novel contact force sensor incorporated in irrigated radiofrequency ablation catheter predicts lesion size and incidence of steam pop and thrombus Circ Arrhythm Electrophysiol 2008 1 5 354 362 10.1161/CIRCEP.108.803650 19808430

[B21] Kuck KH Neuzil P Shah D Herrera C Jais P Saoudi N Hindricks g Kautzner J Lambert H Vivek VY Lack of contact force during RF ablation increases risk for AF recurrences, ESC Congress 2010, Poster 4119

[B22] Weiss S Wirtz D David B Krueger S Lips O Caulfield D Pedersen SF Bostock J Razavi R Schaeffter T In vivo evaluation and proof of radiofrequency safety of a novel diagnostic MR-electrophysiology catheter Magn Reson Med 2011 65 3 770 777 10.1002/mrm.22669 21337409 PMC3715087

[B23] Wittkampf FH Nakagawa H RF catheter ablation: Lessons on lesions Pacing Clin Electrophysiol 2006 29 11 1285 1297 10.1111/j.1540-8159.2006.00533.x 17100685

[B24] Ruffy R Imran MA Santel DJ Wharton JM Radiofrequency delivery through a cooled catheter tip allows the creation of larger endomyocardial lesions in the ovine heart J Cardiovasc Electrophysiol 1995 6 12 1089 1096 10.1111/j.1540-8167.1995.tb00386.x 8720209

[B25] NA 022DKE, Deutsche Kommission Elektrotechnik, Elektrotechnik Informationstechnik in DIN und VDE

[B26] Nordbeck P Hiller KH Fidler F Warmuth M Burkard N Nahrendorf M Jakob PM Quick HH Ertl G Bauer WR Feasibility of contrast-enhanced and nonenhanced MRI for intraprocedural and postprocedural lesion visualization in interventional electrophysiology: animal studies and early delineation of isthmus ablation lesions in patients with typical atrial flutter Circ Cardiovasc Imaging 2011 4 3 282 294 10.1161/CIRCIMAGING.110.957670 21415125

