# Supplementary material for: Direct cooling of the catheter tip increases safety for CMR-guided electrophysiological procedures
Source: J Cardiovasc Magn Reson. 2012 Feb 1;14(1):12. doi: 10.1186/1532-429X-14-12 (PMC3292926; doi:10.1186/1532-429X-14-12)
Supplement: Additional file 1 — Impact of frequency on the power deposition at the catheter tip. The power deposition at the catheter tip into the tissue is dependent on the wave propagation phenomena, distal reflexion and losses in the surrounding medium, and changes with the wave length. This impact of frequency on the power deposition at the catheter tip can be simulated numerically, explaining the rationale behind using two different energy sources (MRI and standard ablation generator) in our test setup. [file 1532-429X-14-12-S1.DOCX]

**Additional file 1: Impact of frequency on the power deposition at the catheter tip**

In this work, the investigation of the MR-induced heat on the one hand and the potential cooling mechanisms of irrigation on the other hand have been investigated using two separate setups. The potential cooling mechanisms were investigated using an energy source for heat production other than the MR scanner’s RF field. This approach is mainly driven by the better practicability of measurements when not operating in an MR environment. Instead of radiating the power into the catheter the experimental setup is designed to galvanically conduct the power into the proximal end of the catheter and an additional neutral electrode. The high frequencies of the MR scanner’s RF field limit the predictability of the actually delivered power at the distal electrode pole. This uncertainty is based on the wave propagation phenomena, distal reflection, and losses in the surrounding medium.

A frequency that is typically used for heat generation during ablation is 500 kHz, and this energy is delivered by an ablation generator. This frequency is preferable for the test setup because at about 500 kHz, the wavelength is still considerably longer than the dimensions of the setup and therefore wave propagation phenomena along the wires and capacitive coupling to the surrounding can be considered negligible.

The area of interest for lesion creation is the interface between tissue and blood. These two media have different electrical characteristics, and the ratio of deposited power in the two media depends on their respective electrical properties. Only the partial power deposited in the tissue is relevant for the assessment of potential tissue damage. This ratio changes with the frequency because the electrical properties of tissue and blood considerably depend on the frequency. The electrical properties of various tissues have been well investigated in the literature over a wide frequency range [1], and the known frequency-dependent change of this power ratio allows compensation when doing the experiments at other frequencies than in the targeted application.

Because the geometry of a catheter tip is already too complex to perform the calculations analytically, a numerical model is employed instead to estimate the energies actually deposited into the tissue at various frequencies. The model represents the distal end of the catheter and its surroundings (blood and tissue), where the catheter is touching the myocardium or pushed into it (Fig. App-1 and Fig App-2). The model is implemented and simulated using Quickfield™ 5.5 taking advantage of its axial symmetry. The tip is modeled as a hemisphere of radius 1 mm that transitions into an isodiametric catheter body on its left side.


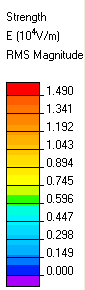

heart

blood

catheter body

tip

*Fig. App-1: FEM model of the catheter with its tip embedded in the myocardium. Colors code the E-field RMS magnitude at 64MHz.*


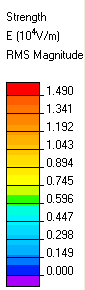

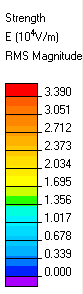


*Fig. App-2: Color coding for the E-field RMS magnitude at 64 MHz (right side) and 500kHz (left side)*

A current of constant magnitude (100 mA – but this value is arbitrary) is injected into the catheter tip. The boundary condition is zero potential on a distant spherical surface. This is reasonable because at larger distances the iso-surfaces of the electric potential are almost spherical. This boundary condition mimics the neutral electrode. Simulations were performed at 500 kHz and 64 MHz. The electrical material properties were chosen according to the frequency (Table 1).

| Frequency (MHz) | Blood | | Heart | |
| --- | --- | --- | --- | --- |
|  | σ (S/m) | ε_r_ | σ (S/m) | ε_r_ |
| 0.5 | 0.748 | 4188 | 0.281 | 3264 |
| 64 | 1.207 | 86.4 | 0.678 | 106 |

*Table 1: Material properties as published by Gabriel [2], where σ is the electrical conductivity and ε_r_ the relative permittivity.*

Three geometrical situations are considered in the simulations:

- The tip only tangentially touches the myocardium (Geometry 1),
- The tip hemisphere is completely inside the myocardium (Geometry 2),
- The tip is pushed 1 mm deeper into the myocardium as compared to Geometry 2 (Geometry 3).

The heat-producing power is only the real part of the complex power values and is the only part considered in the following.

The results are listed below (Fig. App-3 a-c):

| Geometry 1 | Frequency (MHz) | Power in blood (W) | Power in heart (W) | Power fraction in heart |
| --- | --- | --- | --- | --- |
|  | 0.5 | 0.620 | 0.142 | 0.19 |
|  | 64 | 0.313 | 0.097 | 0.24 |
|  | Ratio of the frequency-dependent change in the power fraction in heart |  |  | **1.27** |

Fig. App-3 a) Geometry 1 – The tip touches tangentially the myocardium

| Geometry 2 | Frequency (MHz) | Power in blood (W) | Power in heart (W) | Power fraction in heart |
| --- | --- | --- | --- | --- |
|  | 0.5 | 0.575 | 0.573 | 0.50 |
|  | 64 | 0.216 | 0.300 | 0.58 |
|  | Ratio of the frequency-dependent change in the power fraction in the heart |  |  | **1.16** |

Fig. App-3 b) Geometry 2 – The tip hemisphere is completely inside the myocardium

| Geometry 3 | Frequency (MHz) | Power in blood (W) | Power in heart (W) | Power fraction in heart |
| --- | --- | --- | --- | --- |
|  | 0.5 | 0.131 | 1.258 | 0.91 |
|  | 64 | 0.058 | 0.457 | 0.89 |
|  | Ratio of the frequency-dependent change in the power fraction in heart |  |  | **0.98** |

Fig. App. 3 c) Geometry 3 – The tip is pushed 1mm deeper into the myocardium as compared to Geometry 2

It can be concluded that the worst-case scenario is Geometry 1, where the power injection at 500 kHz would underestimate the power deposited in the heart tissue (and thus the resulting temperature rise) by 27%. However, in this case the absolute power affecting the heart is only in the range of approx 20% of the total injected power. The highest error occurs when the risk is minimal. For Geometry 2 the error is reduced to 16%. Geometry 3 is quite close to the homogeneous case because the tissue-blood interface is quite far from the tip. Therefore, its field shape is no longer significantly affected. There is almost no frequency-dependent error in this case where the tip is embedded only 1 mm deeper into the tissue.

According to the protocol used in this study, this frequency-dependent error is avoided to the greatest possible extent because the temperature itself is the parameter that is adjusted in the bench setup to fit to the measurements in the MR scanner setup. A smaller deposited power is compensated by using a higher power output at 500 kHz. Consequently, the power deposited inside the tissue is similar in both setups, allowing a direct comparison of the measured temperatures.

1. Gabriel S, Lau RW, Gabriel C: **The dielectric properties of biological tissues: II. Measurements in the frequency range 10 Hz to 20 GHz**. *Phys Med Biol* 1996, **41**(11):2251-2269.

2. C. Gabriel, S. Gabriel. Compiliation of the Dielectric Properties of body tissues at RF and microwave frequencies. 1996, last updated 1997; <http://niremf.ifac.cnr.it/docs/DIELECTRIC/Report.html>
